# A Cost-Benefit Analysis of Alternative Management Strategies for Red Deer in Denmark

**DOI:** 10.1007/s00267-024-02023-y

**Published:** 2024-08-07

**Authors:** Frank Jensen, Thomas Lundhede, Peter Sunde

**Affiliations:** 1 Department of Food and Resource Economics, University of Copenhagen, Rolighedsvej 23, 1958 Frederiksberg C, Denmark; 2 Department of Agricultural Economics, Extension and Rural Development, University of Pretoria, Pretoria, South Africa; 3 Department of Ecoscience – Wildlife Ecology, Aarhus University, C.F. Møllers Alle 8, 8000 Aarhus C, Denmark

**Keywords:** Cost-benefit analysis, Sex and age-structured population model, Management strategies for red deer in Denmark, D61, Q29, Q51

## Abstract

In this paper, we conduct a cost-benefit analysis (CBA) of five alternative management strategies for red deer in Denmark: free harvest, trophy hunting, maximum harvest and two cases for natural demographic population compositions. To capture the outcome under each strategy we use a biological sex- and age-structured population model. The net benefit function includes meat values, recreational values, browsing damage costs and traffic damage costs and these values and costs are assumed to differ for the various sex and age classes of red deer. We show that the maximum harvest strategy leads to a reasonably high positive total net benefit, while the free harvest strategy yields a small positive net benefit. On the other hand, the trophy hunting strategy generates a high negative net benefit, while small negative net benefits are obtained under the two strategies for natural demographic population compositions.

## Introduction

A cost-benefit analysis (CBA) can be used to evaluate various management strategies in terms of economic efficiency (see, e.g., Mishan and Quah, 2021). Specifically, CBAs have been used to investigate management strategies within several policy areas, such as the environment, health and traffic (see, e.g., Edge, 2021). Within environmental economics, CBA is a well-established approach to evaluate strategies to protect and manage renewable natural resources, with urban forest and marine ecosystems as examples (see, e.g., Song et al., 2018 and Sumaila, 2004). It is important to distinguish between two types of CBAs. First, a CBA can be used to evaluate strategies that directly affect the resource allocation in a society (see, e.g., Mishan and Quah, 2021). This can be labeled a conventional CBA, and here, the benefits and costs under various strategies are measured and compared directly (see, e.g., Pearce, 1983). Examples of policy issues related to animal species which have been evaluated with a conventional CBA is rehabilitee centers in Catalonia (see Molina-Lopez et al., 2017) and mitigation measures for reducing collisions with motor vehicles in United States and Canada (see Huijser et al., 2009).

Second, a CBA can be used to evaluate the effect of changes in values of regulatory instruments (see, e.g., Baldwin et al., 2018). A change in a regulatory instrument will affect the decisions by private agents, and it is this change that generate benefits and costs. To predict the response by private agents, either a partial or general equilibrium model can be used (see, e.g., Farrow and Rose, 2018) and based on the results from such models the benefits and costs can be calculated and compared. This approach can be denoted a CBA of changes in a regulatory instrument. As examples for animal species, Reyns et al. (2018) have used a partial equilibrium model to conduct a CBA of invasive species control in the Flanders while Shwiff et al. (2016) have undertaken a CBA of mitigation strategies for disease transmission among wildlife and livestock populations in Mexico by using a general equilibrium model.

Following Svensson et al. (2015) the total net benefit of many animal species exposed to hunting can be defined as meat and recreational values corrected for damage costs related to browsing and traffic incidents (browsing and traffic damage costs). However, it is important to distinguish between economically optimal and nonoptimal management strategies (see, e.g., Milner-Gulland et al., 2004). Under an optimal strategy, the net benefit is maximized subject to restrictions generated by the population of animal species (see, e.g., Clark, 1990). By solving this problem, we obtain an economically optimal population and harvest of a species and this can be defined as an optimal management strategy. When conducting a CBA an optimal strategy we obtain with the highest possible net benefit since this net benefit is maximized (see, e.g., Olaussen and Skonhoft, 2011).

However, targeting an optimal population and harvest of animal species is not always politically feasible. From a practical policy perspective it can, therefore, be necessary to fix a nonoptimal population and harvest and this can be defined as a nonoptimal management strategy (see, e.g., Svensson et al., 2015). From an economic perspective it is commonly argued, that we should aim for achieving the highest possible net benefit given the nonoptimal population and harvest. Therefore, we shall undertake a CBA of a nonoptimal strategy (see, e.g., Svensson et al., 2015).

For many species, nonoptimal strategies are formulated as restrictions on hunting, which will affect the population (see, e.g., Garshelis et al., 2020). For predicting the effect of various hunting strategies on the population we can use a biological population model. Thus, we can identify the population and harvest under various nonoptimal strategies and thereby the net benefit. For a CBA of restrictions on hunting we can therefore use the same approach as for a change in the value of regulatory instruments.

However, meat values, recreational values, browsing damage costs and traffic damage costs may differ for various sex and age classes of animal species (see, e.g., Rondeau and Conrad, 2003 and Olaussen and Skonhoft, 2011). Thus, the net benefit will differ between various sex and age classes of animal species. Furthermore, nonoptimal strategies are often formulated as separate hunting restrictions for each sex and age class (see, e.g., Svensson et al., 2015). Thus, to identify the population and harvest under nonoptimal strategies it can be useful to use a sex- and age-structured biological model. Red deer is an example of an animal species for which values and costs may differ for various sex and age classes (see, e.g., Skonhoft et al., 2013) and it can therefore be argued that nonoptimal management strategies should differ for various sex and age classes (see, e.g., Smart et al., 2008). To conduct a CBA of such strategies for red deer we may then choose to consider to use a sex- and age-structured biological model.

Several papers have used sex- and age-structured biological models to investigate economically optimal management strategies for red deer. Specifically, by using a sex and age-structured model Skonhoft et al. (2013) consider optimal harvest levels for red deer in Norway, Smart et al. (2008) identify an optimal management strategy for red deer in the Scottish highlands, and Rondeau and Conrad (2003) investigate optimal management of urban deer in a geographical region in the United States. More importantly, a sex-structured model is used to conduct a CBA of various nonoptimal strategies for harvesting red deer in Scotland by Milner-Gulland et al. (2004), but only meat values are taken into account. Thus, to our knowledge, there has been no attempt to use a sex- and age-structured biological model to undertake a CBA of nonoptimal strategies for harvesting red deer where meat values, recreational values, browsing damage costs and traffic damage costs of traffic is included.

However, an advanced sex- and age-structured biological model is often very complicated and for undertaking a CBA it is useful to impose two simplifying assumptions. First, it is reasonable to restrict attention to a static, steady-state equilibrium implying that the population, harvest and net benefit for each sex and age class is constant over time. Of course it would be desirable to use a dynamic sex- and age-structured model and allow for adjustment paths owards a steady-state equilibrium. This implies that the population, harvest and net benefit for each population category may change over time (see, e.g., Clark, 1990). However, it is well-known that dynamic sex- and age-structured models may generate strange adjustment paths even under simplifying assumptions (see, e.g., Quass and Tahvonen, 2019). Therefore, it is reasonable to use a static sex- and age-structured model.

Second, to compare alternative nonoptimal management strategies using a biological model it is convenient to assume an identical initial population under each strategy. Thus, we conduct a CBA of the composition of the population and harvest on various sex and age classes. Within a sex- and age-structured model, both the size and composition of the population and harvest is important for the net benefits under each strategies (see, e.g., Olaussen and Skonhoft, 2011). However, to explain the results of a CBA it is useful to focus on one effect and we have chosen to consider the composition (and not the size) of the population and harvest.

In this paper, we conduct a CBA of alternative nonoptimal management strategies for red deer in Denmark using a sex- and age-structured biological model from Sunde and Haugaard (2014) who assume that the population and harvest of each sex and age class is in a steady-state equilibrium. The initial population under each strategy is fixed to 1000 individuals implying that we undertake a CBA of the composition of the population and harvest on sex and age classes under various nonoptimal management strategies. The population and harvest of red deer are structured into female calves, female yearlings, adult females, male calves, male yearlings, young stags, near-mature stags and mature stags. In the net benefit function we include meat values, recreational values, browsing damage costs and traffic damage costs and these values and costs are assumed to differ for the various sex and age classes of red deer. We investigate five nonoptimal hunting strategies represented by free harvest, trophy hunting, maximum harvest and two cases for natural demographic population compositions. For each strategy, we calculate the net benefit, and from an economic point of view, we should implement the strategy that generates the highest net benefit.

The rest of the paper is organized as follows. In Section “Biological model and management strategies”, we will briefly introduce the sex- and age-structured biological model and management strategies while the net benefit function is defined in Section “Annual net benefit”. The Section “Parameterization for red deer in Denmark” contains a description of the approaches we have used to identify relevant values and costs for red deer in Denmark and in Section “Results”, we present the results of the CBA. A brief summary and discussion can be found in Section “Brief summary and discussion”.

## Biological Model and Management Strategies

### The Model

The sex- and age-structured population model is available as a sex-specific lifetable with 25 age classes where the demographic composition of the population and number of individuals dying are the emergent pattern of: (a) age-specific fecundity; (b) the proportion of recruits entering the population that are females; and (c) age-specific mortality of males and females (see Caughley, 1977). The model is static in the sense that the number of deaths equals the number of individuals born every year (no emigration or immigration). Hence, the population is stable over time and in a steady-state equilibrium. To ensure a steady-state equilibrium we have only used combinations of age-specific fecundity and mortality rates, which secure that the number of births is equal to the number of deaths. A detailed description and a copy of the model (with the results under all five management scenarios) can be found in appendixes which are available as online supplementary material.

Now we will make a mathematical characterization of the main components of the model. We let ${x}$ denote the age of red deer (${x}$ = 0, …, 24) and the number of calves (of 0 years old) recruited to the population each year (${n}_{0}$) can be defined as:

1 $${n}_{0}=\mathop{\sum }\limits_{x=1}^{24}{n}_{x,f}{m}_{x}$$

where ${n}_{x,f}$ is the number of females in age class ${x}$ and ${m}_{x}$ is age-specific fecundity rates. To estimate the age-specific fecundity rates, which enters in Eq. (1), we used Danish population data on the proportion of lactating hinds at the start of the hunting season, which is 1st September. Thereby, we obtain that ${m}_{x}=$0.57 for females of 1 years old and ${m}_{x}=$0.82 of females of 2 + years old (see Sunde and Haugaard, 2014). As red deer are polygynous, we did not consider the proportion of males in the population as important for fecundity rates as long as there is minimum one adult male for every 20 females of 1+ years old (see Mysterud et al., 2002).

The number of females (*f*) and males (*m*) recruited to the population (${n}_{0,f}$ and ${n}_{0,m}$) can be defined as:

2 $${n}_{0,f}={n}_{0}{P}_{f}$$

3 $${n}_{0,m}={n}_{0}(1-{P}_{f})$$

where ${P_f}$ is the proportion of calves which are females. In relation to Eqs. (2) and (3) we assumed that ${P_f}$  = 0.5 implying that half of all recruits are females while the other half are males.

The proportion of calves which survives to age ${x}$ $({l}_{x}=\frac{{n}_{x}}{{n}_{0}})$ can be defined as the product of age-specific survival rates for the preceding age classes. By distinguishing between females and males we get that:

4 $$\begin{array}{cc}{l}_{x,f}=\frac{{n}_{x,f}}{{n}_{0,f}}=(1-{q}_{O,f})\ast {.......}.\ast (1-{q}_{x-1,f}) & {\rm{for}}\,x=1,\ldots \mathrm{.}.,24\end{array}$$

5 $$\begin{array}{cc}{l}_{x,m}=\frac{{n}_{x,m}}{{n}_{0,m}}=(1-{q}_{O,m})\ast {.......}.\ast (1-{q}_{x-1,m}) & {\rm{for}}\,x=1,\ldots \mathrm{.}.,24\end{array}$$

where ${q}_{x,f}$ and ${q}_{x,m}$ is age-specific mortality rate while $1-{q}_{x,f}$ and $1-{q}_{x,m}$ is age-specific survival rates for females and males, respectively. By using Eqs. (4) and (5) the total number of females and males surviving to age ${x}$ (${n}_{x,f}$ and ${n}_{x,m}$) become:

6 $$\begin{array}{cc}{n}_{x,f}={n}_{0,f}{l}_{x,f} & {\mathrm{for}}\,x=1,\ldots {.}.,24\end{array}$$

7 $$\begin{array}{cc}{n}_{x,m}={n}_{0,m}{l}_{x,m} & {\mathrm{for}}\,x=1,\ldots {.}.,24\end{array}$$

From Eqs. (6) and (7) it follows that the age-specific mortality rates affects the population of males and females in each age class (*x*) since these rates enters in ${l}_{x,f}$ and ${l}_{x,m}$ which is defined in Eqs. (4) and (5). In this paper ${n}_{x,f}$ and ${n}_{x,m}$ is denoted the population of females and males at age class *x*.

By using Eqs. (6) and (7) the number of females and males dying in each age class ($d{n}_{x,f}$ and $d{n}_{x,m}$) is:

8 $$\begin{array}{cc}d{n}_{x,f}={n}_{x,f}{q}_{x,f} & {\rm{for}}\,x=1,\ldots {.}.,24\end{array}$$

9 $$\begin{array}{cc}d{n}_{x,m}={n}_{x,m}{q}_{x,m} & {\mathrm{for}}\,x=1,\ldots \mathrm{.}.,24\end{array}$$

From Eqs. (8) and (9) it follows that the age-specific mortality rates affect the number of females and males dying in two ways. First, the age-specific mortality rates enter directly in Eqs. (8) and (9). Second, the population of females and males, which enter in Eqs. (8) and (9), depend on ${l}_{x,f}$ and ${l}_{x,m}$ (see Eqs. (6) and (7)) which depend on the age-specific mortality rates as can be seen from Eqs. (4) and (5).

In managed populations, sex- and age- specific mortality consist of management steered deaths (harvest) and other causes (natural causes and traffic). Given the population is managed below carrying capacity, the population surplus must be harvested to keep the population stable. In the model we allow for a baseline mortality rate for all sex and age classes on 0.02 caused by vehicle collisions (see the subsection “Probability of a vehicle collision”). For simplicity we assume that the population is entirely regulated by humans, which means that all mortality is assumed to be due to hunting (apart from the baseline mortality for all sex and age classes caused by vehicle collisions). This implies that we have ignored predation and other kinds of natural mortality, and we will therefore operate with hunting mortality rates in this paper. Even though vehicle collisions do not always result in the death of red deer, we assume that all individuals involved in vehicle collisions die. Therefore, we let the vehicle collision rate capture all other causes of deaths apart from hunting. One implication of this is that $d{n}_{x,f}$ and $d{n}_{x,m}$ in Eqs. (8) and (9) becomes equal to the total harvest of females and males of each age class (apart from the mortality due to vehicle collisions). from above it follows that, the total harvest of each sex and age class will depend on the sex- and age-specific hunting mortality rates.

The total population at the start of the hunting season (${N}_{September}$) and in spring (${N}_{spring}$) (after the hunting season but before calves is born) is defined as the sum of all males and females in all age classes:

10 $${N}_{September}=\mathop{\sum }\limits_{x=0}^{24}{n}_{x,f}+\mathop{\sum }\limits_{x=0}^{24}{n}_{x,m}$$

11 $${N}_{,Spring}=\mathop{\sum }\limits_{x=1}^{24}{n}_{x,f}+\mathop{\sum }\limits_{x=1}^{24}{n}_{x,m}$$

Under all management strategies described below the spring population is scaled to consist of 1000 individuals implying that we consider the composition (and not the size) of the population on various sex and age classes. Furthermore, in Eqs. (10) and (11) we have that ${N}_{September}$ is equal to ${N}_{Spring}$ plus the number of calves born during the summer and surviving to the start of the hunting season. From Eq. (1) it is clear that ${N}_{September}$ varies between management strategies because of variations in the number of females of 1+ years old.

### Management Strategies

We use the biological population model to capture five different nonoptimal management strategies for the population and harvest of red deer in Denmark. An overview of the strategies is provided in Table 1.

**Table 1:** Nonoptimal management strategies for red deer in Denmark

Strategy	Label	Description
**1**	**Free harvest**	Unrestricted harvest within a hunting season
**2**	**Trophy hunting**	Harvest as many mature males as possible
**3**	**Maximum harvest**	Harvest as many individuals as possible
**4.A**	**Natural demographic population composition**	Demographic distribution that is natural for red deer populations (identical hunting mortality rates for mature males)
**4.B**	**Natural demographic population composition**	Demographic distribution that is natural for red deer populations (differentiated hunting mortality rates for mature males)

As mentioned in subsection “The model” when using the biological model, different hunting mortality rates for calves, females and males of each age class result in different compositions of the population. Therefore, the population of each sex and age class (including calves) depend on the hunting mortality rates. Each management scenario is therefore characterized by different assumptions about sex- and age-specific hunting mortality rates. Since it is difficult for hunters to identify the sex of calves, we assume an identical calf hunting mortality rate for females and males. Furthermore, we assume that hunters are unable to estimate the age of females, implying that the hunting mortality rate is identical for all females of 1+ years old. We assume that hunters can estimate the age of mature males; therefore, age-specific hunting mortality rates of males is therefore possible.

In each of the following subsections, we will describe each management strategy.

#### Free Harvest (Strategy 1)

This strategy captures the management regime in Denmark before 2017, when initial restrictions on harvest of males within the hunting season were introduced in some regions. Under the free harvest strategy, the sex- and age-specific hunting mortality rate is assumed to be 0.35 for females and males of 0 years old, 0.20 for females of 1+ years old, 0.35 for males of 2 and 3 years old and 0.50 for males of 4+ years old. Under this strategy, approximately 1% of all males survive to become 8+ years old, which is in accordance with population data for red deer in Denmark (see Sunde and Haugaard, 2014).

#### Trophy Hunting (Strategy 2)

The objective under this strategy is to harvest as many mature males as possible, and this is accomplished by letting as many males as possible survive until they become old. As males and females of 0 years old cannot be unambiguously distinguished, the hunting mortality rate for both is assumed to be 0.35, while we assume that the hunting mortality rate for females of 1+ years old is 0.20. For males of 1 to 7 years old the hunting mortality rate is assumed to be 0. For males of 8+ years old, free hunting is allowed but we assume that the hunting mortality rate is 0.50 since it is unrealistically to harvest all mature males. Approximately 56% of all males survive to become 8+ years old under this strategy.

#### Maximum Harvest (Strategy 3)

Under this strategy we want to maximize the number of harvested individuals implying that the population should consist of as many females as possible to increase the recruitment. The number of mature males should be kept as low as possible, but to secure conception, there must be 1 mature male for every 20 females (see Mysterud et al., 2002). Under the maximum harvest strategy, the hunting mortality rate is assumed to be 0.72 for females and males of 0 years old, 0.10 for females of 1+ years old, 0.77 for males of 1 years old, 0.02 for males of 2 to 7 years old and 0.50 for males of 8+ years old. With the maximum harvest strategy between 5% and 6% of all males survive to become 8+ years old.

#### Natural Demographic Population Compositions (Strategies 4.A and 4.B)

With these two strategies, we want to achieve demographic compositions similar to naturally regulated populations. In such populations, the hunting mortality is high for red deer of 0 years old, at its lowest for animals between 1 and 8–10 years old and gradually increasing for red deer older than 8–10 years (see Clutton-Brock et al. 2002, Lowe, 1969 and Wright et al., 2006). To achieve a higher share of old males, a governmental management objective in Denmark is that these shall comprise at least 5% of all individuals in the spring population. We present two alternative strategies under which natural demographic compositions can be achieved. Under strategy 4.A, we assume an identical hunting mortality for males of 1+ years old. Thus, we assume that the hunting mortality rate is 0.50 for females and males of 0 years old, 0.163 for females of 1+ years old, and 0.26 for males of 1+ years old. With strategy 4.B, we assume that hunting mortality rates for males are differentiated. Specifically, the hunting mortality rate is assumed to be 0.50 for females and males of 0 years old, 0.163 for females of 1+ years old, 0.20 for males of 2 to 7 years old and 0.40 for males of 8+ years old. Under strategy 4.A and 4.B, 6.1% and 10.5% of all males survive to become of 8+ years old respectively.

### Sex and Age Classes

To conduct the CBA we must decompose the population and harvest of red deer found by using the biological model on various sex and age categories. In Table 2, we have summarized the relevant sex and age classes together with a label used to characterize each category.

**Table 2:** Sex and age classes for red deer in Denmark

Category	Label	Description
**Female calves**	FC	Females in their first year of life (0 years old)
**Female yearlings**	FY	Females in their second year of life (1 years old)
**Adult females**	FA	Females in their third year of life or older (2+ years old)
**Male calves**	MC	Males in their first year of life (0 years old)
**Male yearlings**	MY	Males in their second year of life (1 years old)
**Young stags**	YS	Males between three and five years of life (2 to 4 years old)
**Near-mature stags**	NS	Males between their six and eight years of life (5 to 7 years old)
**Mature stags**	MS	Males in their ninth year of life or older (8+ years old)

For calves (0 years old), we distinguish between females and males since the body weight is slightly higher for the latter (see Skonhoft et al., 2013 and Sunde and Haugaard, 2014). We also include female yearlings (1 years old) because the body weight of this category is higher than for female calves (see Skonhoft et al., 2013). Furthermore, during the summer, 57% of all female yearlings give birth to a calf which survives until the start of the hunting season (see Sunde and Haugaard, 2014). In addition, we introduce adult females (2+ years old) since this age class is heaviest and most fertile (see Skonhoft et al., 2013). Specifically, 82% of all adult females give birth to a calf before the start of the hunting season (see Sunde and Haugaard, 2014). Male yearlings (1 years old) are also included since this category has a significantly higher body weight than male calves. Furthermore, we introduce young stags (2 to 4 years old) since this age category is heavier than younger males, although they have not yet reached the maximum size (see Skonhoft et al., 2013). We also operate with near-mature stags (5 to 7 years old) since this category has a size of antlers and a body size which is reasonably close to its maximum (see Skonhoft et al., 2013). There are relatively few near-mature stags in Denmark and most hunters consider them as fully mature (see Sunde and Haugaard, 2014). Finally, we operate with mature stags (8+ years old) since the size of the antlers and the body weight for this category are at their maximum (see Skonhoft et al., 2013). In Denmark mature stags are very rare in unfenced populations due to a high hunting pressure on stags.

## Annual Net Benefit

The results from the biological model have been used to can obtain the population and harvest for each sex and age classes (see Table 2) under each nonoptimal strategy (see Table 1). Since we consider nonoptimal population and harvest levels, we can also calculate the net benefit under each strategy. We follow Svensson et al. (2015) and in the benefit function we have included meat values, recreational values, browsing damage costs and traffic damage costs. By using the labels from Table 2, the annual net benefit becomes:

12 $$\begin{array}{c}NB=m({w}_{FC}{h}_{FC}+{w}_{FY}{h}_{FY}+{w}_{FA}{h}_{FA}+{w}_{MC}{h}_{MC}+{w}_{MY}{h}_{MY}+{w}_{YS}{h}_{YS}+{w}_{NS}{h}_{NS}+{w}_{MS}{h}_{MS})\\ +{r}_{FC}{h}_{FC}+{r}_{FY}{h}_{FY}+{r}_{FA}{h}_{FA}+{r}_{MC}{h}_{MC}+{r}_{MY}{h}_{MY}+{r}_{YS}{h}_{YS}+{r}_{NS}{h}_{NS}+{r}_{MS}{h}_{MS}-\\ {b}_{FC}{n}_{FC}-{b}_{FY}{n}_{FY}-{b}_{FA}{n}_{FA}-{b}_{MC}{n}_{MC}-{b}_{MY}{n}_{MY}-{b}_{YS}{n}_{YS}-{b}_{NS}{n}_{NS}-{b}_{MS}{n}_{MS}-\\ \alpha ({t}_{FC}{n}_{FC}+{t}_{FY}{n}_{FY}+{t}_{FA}{n}_{FA}+{t}_{MC}{n}_{MC}+{t}_{MY}{n}_{MY}+{t}_{YS}{n}_{YS}+{t}_{NS}{n}_{NS}+{t}_{MS}{n}_{MS})\end{array}$$

where ${NB}$ is the total annual net benefit, ${h_i}$ (for ${i}$  = *FC, FY, FA, MC, MY, YS, NS* and *MS*) is the harvest (of each sex and age class), ${n_i}$ is the population, ${m}$ is a constant price on red deer meat (assumed to be identical for all sex and age classes), ${w_i}$ is the weight of harvested red deer, ${r_i}$ is a constant marginal recreational value, ${b_i}$ is a constant marginal browsing damage cost, ${t_i}$ is a constant reflecting the marginal traffic damage costs, and ${\alpha}$ is a probability for one red deer to be involved in a traffic incidents (assumed to be constant and identical for all sex and age classes) which captures that only a part of a population of red deer is involved in traffic incidents.

Note five facts in relation to Eq. (12). First, if we want to identify an optimal management strategy (an optimal population and harvest for each sex and age class) we should maximize Eq. (12) (subject to restrictions for the population of each category). However, want to evaluate various nonoptimal strategies (a nonoptimal population and harvest) and, therefore, we can calculate and compare the net benefit under each strategy by using Eq. (12).

Second, the meat and recreational values are assumed to depend on the harvest (of each sex and age class) of red deer, while the browsing and traffic damage costs are assumed to be related to the population. These functional forms can be discussed, but similar assumptions are adopted in other economic studies of the management of animal species exposed to hunting (see, e.g., Olaussen and Skonhoft, 2011). Note that a high trophy value is captured by a large marginal recreational value for mature stags in Eq. (12).

Third, we assume constant marginal values and costs (of each sex and age class) of red deer. Specifically, $m{w}_{i}$ is a constant marginal meat value, ${r}_{i}$ is a constant marginal recreational value, ${b}_{i}$ is a constant marginal browsing damage cost and $\alpha {t}_{i}$ is a constant marginal traffic damage cost. It can be discussed whether the marginal values and costs are constant, and an alternative is to assume that the marginal values is positive but decreasing in the harvest, while the marginal costs are positive and increasing in the population (see, e.g., Dietz and Hepburn, 2010). However, assuming nonconstant marginal values and costs will cause problems for parameterization of the model for red deer in Denmark. Thus, as a simplification, we assume constant marginal values and costs.

Fourth, Eq. (12) can be used to express various decision rules for management strategies of red deer in Denmark. Assume, first, that we only have one strategy which is denoted ${j}$. Now, $N{B}_{j} > 0$ implies that the strategy shall be implemented, while $N{B}_{j} < 0$ indicates that a strategy shall not be implemented (see, e.g., Mishan and Quah, 2021). However, we can say more about a strategy where $N{B}_{j} < 0$. From Eq. (12), we have that if $N{B}_{j} < 0$, then the total browsing and traffic damage costs are higher than the total meat and recreational value. Furthermore, Eq. (12) implies that $NB=0$ if the population and harvest for all sex and age classes are zero, and this is preferred over a strategy under which $N{B}_{j} < 0$. A population and harvest on zero for all sex and age classes imply that the population of red deer shall be extinct. Thus, if $N{B}_{j} < 0$, it is optimal to drive the red deer population to extinction to avoid the browsing and traffic damage costs (see, e.g., Sinden, 2019). Next, assume that we have two (or more) strategies which is denoted ${j}$ and ${k}$. Now, if $N{B}_{j} > N{B}_{k}$ and $N{B}_{j} > 0$ strategy ${j}$ shall be implemented, while $N{B}_{k} > N{B}_{j}$ and $N{B}_{k} > 0$ imply that we shall select strategy ${k}$ (see, e.g., Mishan and Quah, 2021). Finally, if $N{B}_{j} < 0$ and $N{B}_{k} < 0$ the population of red deer shall be driven toward extinction, since this implies that $NB=0$ (see, e.g., Sinden, 2019). These decision rules will be used to investigate the nonoptimal management strategies for red deer in Denmark summarized in Table 1.

Finally, in a CBA, it is common to define *NB* Use NB instead and write it as a mathematical expression as the present value of the current and future; therefore, a discount rate (or factor) must be taken into account (see, e.g., Mishan and Quah, 2021). However, the biological model is based on an assumption about a steady-state equilibrium implying that the population, harvest and net benefit are constant over time. Furthermore, we only want to investigate which of the strategies in Table 1 to implement. Thus, we can restrict attention to the annual net benefit defined in Eq. (12), implying that future time periods and discounting can be disregarded. Specifically, if the annual net benefit is positive and higher under strategy *j* compared to strategy *k*, the discounted net benefit is also higher in the former case. Furthermore, if a strategy yields a negative annual net benefit, the discounted net benefit will also be negative. Taking several time periods into account will therefore only affect the size of the net benefits under each scenario but not the ranking (see, e.g., Daly, 1974).

## Parameterization for Red Deer in Denmark

To calculate the annual net benefit in Eq. (12) for each nonoptimal strategy summarized in Table 1, we need parameter estimates for the components of the marginal values and costs for red deer in Denmark. Table 3 provides an overview over the marginal values and costs.

**Table 3:** Annual benchmark marginal values and costs for each sex and age class for red deer in Denmark

		Price on red deer (DKK per kg)	Weight of red deer (kg per individual)	Marginal recreational value (DKK per individual)	Marginal browsing damage costs (DKK per individual)	Constant reflecting marginal traffic damage costs (DKK per individual)	Probability of a traffic incident
**Valuation method**		Market price	Calculation	Unadjusted benefit transfer	Adjusted benefit transfer	Unadjusted benefit transfer	Calculation
**Nature of price**		Consumer		Consumer	Producer	Producer	
**Year for price**		2022		2022	2020	2019	
**Parameter values**	**Population category**						
	Female calves	20	38.3	3182	564	12,423	0.02
	Female yearlings	20	60.0	3391	898	19,787	0.02
	Adult females	20	73.9	3600	1094	24,101	0.02
	Male calves	20	40.7	3182	607	13,364	0.02
	Male yearlings	20	64.5	3746	950	20,922	0.02
	Young stags	20	113.6	5130	1677	36,946	0.02
	Near-mature stags	20	131.6	6117	1939	42,720	0.02
	Mature stags	20	118.7	7272	1818	40,060	0.02

In Table 3 all marginal values and costs covers one year and represent benchmark parameter estimates. More importantly, we have used simple methods such as benefit transfer to estimate the benchmark parameter values (see, e.g., Johnston et al., 2015 for an introduction to benefit transfer). This implies that the marginal values and costs are highly uncertain and to minimize this uncertainty we have used other studies for red deer in Denmark to undertake benefit transfer. Furthermore, we have conducted sensitivity analyses and the result of these are presented in subsection “Sensitivity analyses” below. In a sensitivity analysis, it is common to vary each benchmark parameter value separately while keeping the other parameters at the benchmark level. However, to ensure consistency with the ranking of the marginal values and costs in Table 3 for the various sex and age classes, we chose to vary the benchmark parameter estimates for the same marginal value or cost simultaneously. As an example, we have varied the marginal browsing damage cost for all sex and age classes while keeping all other marginal values and costs at the benchmark level. We have chosen to vary the benchmark estimates by ± 50% and a decrease in a set of parameter values is denoted a lower bound, while an increase represents an upper bound.

In Table 3 we have also indicated whether a marginal value or cost is measured by consumer or producer prices (nature of price). In a CBA, all marginal values and costs should be measured in identical prices, and we use producer prices (see, e.g., Mishan and Quah, 2021). Thus, we have corrected marginal values and costs in consumer prices for a value added tax. In Table 3 we have also indicated the year for measuring a marginal value and cost (year for price). It is well-known that all marginal values and costs should be measured in prices for the same year (see, e.g., Mishan and Quah, 2021). In this paper, we use prices for 2022 and we have adjusted marginal values and cost measured for other years using a net price index for Denmark (see Statistics Denmark, 2023a).

In each subsection below we will verbally describe how the relevant marginal values and costs have been identified.

### Meat Price

As a measure for meat price, we have identified the willingness-to-pay (WTP) for red deer meat in Denmark using a constant market price. We assume no significant difference in the quality of red deer meat between sex and age classes, implying that the meat price is assumed to be identical for all population categories. However, access to game meat is not common in Denmark unless you are a hunter, implying that an official market for unprocessed red deer meat does not exist. Therefore, we have used a price on red deer meat on an informal market in Denmark, where hunters sell meat before any processing except gutting. The price on the informal market for all parts of a red deer was approximately 20 DKK/kg in 2022.^1^

### Weight of Red Deer

To estimate the weight of red deer in Denmark, we use sex- and age-specific data reported by hunters between 2008 and 2013 for Djursland, which is a region in Denmark (see Sunde and Haugaard 2014). We use the weight of an entire animal without entrails, which is consistent with the fact that the market price covers red deer meat before any processing except gutting (see subsection “Meat price”).^2^

### Marginal Recreational Value

The recreation value of red deer in Denmark should ideally include three components: (a) the recreational use value of hunters participating in hunting; (b) the recreational use value for watching red deer by the general public; and (c) the existence value of the general public of knowing that a population of red deer exists. However, the relationship between a population of red deer and recreational and existence values of the general public is unknown. Thus, it is difficult to obtain a reliable measure for (b) and (c). Furthermore, for red deer in Denmark (b) and (c) are probably low compared to (a) (see Kanstrup et al., 2009). Therefore, we only include recreational use values for hunters in this paper. To estimate the marginal recreational value for red deer in Denmark, we conduct an unadjusted benefit transfer (see, e.g., Johnston et al., 2015). Specifically, we use an estimate for the marginal recreational value from Jensen et al. (2022), who have examined the market for red deer hunting in Denmark.^3^ The utility of recreational hunting depends on the size of the animal bagged, but a number of studies indicate that time spent on hunting also matters (see Aiken and Rouche, 2001 and Boman et al., 2011). Thus, by using the market prices for trophies and the number of hunting days in Jensen et al. (2022), we are able to distribute the marginal recreational value to our sex and age classes (see Table 2). Note also that the marginal recreational values in Table 3 are higher for near-mature stags and mature stags than for the other population categories. This result arise because the market price for trophies is used to allocate the marginal recreational value to our sex and age classes. In this way, we have accounted for a trophy value related to harvesting red deer.

### Marginal Browsing Damage Costs

Browsing damage cost on agricultural land arises when a population of red deer forage on crops or alternatively rest on or pass crops in the fields. A population of red deer will also affect forest products since browsing (or alternatively rubbing of antlers) injures trees and generates losses of volume and/or quality of timber (see MacMillan and Leitch, 2008, Ward et al., 2004 and Nørgaard, 2014). However, due to lack of data for Denmark, we have not included the browsing damage costs on trees and several studies indicate that the browsing damage cost on agricultural land is much higher than the browsing damage cost on trees (see, e.g., Skonhoft et al., 2013 and Thorvaldsen et al., 2010). To identify the browsing damage cost on agricultural crops for red deer in Denmark, we undertake an adjusted benefit transfer by using a measure from Løbner (2021), who has conducted a survey among farmers in the west and central part of Jutland in Denmark. In the survey, farmers were asked to self-report the actual crop damage costs caused by red and fallow deer in 2020. We have adjusted this self-reported cost measure by using the share of crop damage caused by fallow deer. However, a potential problem with using self-reported browsing costs is that farmers may have an incentive to exaggerate these if they believe that this can affect future compensations (see, e.g., Rollins and Briggs, 1996). A relatively large share of the farmers, who were invited to participate in the survey, did not respond. We assume that the browsing damage cost for these farmers is zero, and by adjusting with the average farm sizes for respondents who participate in the survey, a measure for the browsing damage cost per hectare can be obtained. By using information for 2020 about agricultural land and crop types from Statistics Denmark (2023b), the area under cultivation can be estimated. For the west-central part of Denmark, where the survey in Løbner (2021) was carried out, there is approximately 350,000 hectares of land under cultivation, and this area can potentially be affected by browsing damage from red deer. By using this area, the total damage browsing cost on agriculture crops can be estimated.

To calculate the browsing damage cost per individual (the marginal browsing damage cost), we need information about the population of red deer for the western and central parts of Jutland where the crop damage is reported. The population of red deer in 2020 can be estimated using $N=CL\lambda /\eta$, where ${C}$ is the number of red deer reported culled during a hunting season, ${L}$ is the mean age of harvested red deer (longevity), ${\eta}$ is the proportion of all deaths due to culling in the population while ${\rm{\lambda}}$ is an annual growth rate. According to the Danish bag statistics (available at https://fauna.au.dk/), a total of 3612 individuals of red deer were culled in the relevant area in the hunting season for 2020/21. By using the number of deer killed in vehicle collisions, the proportion of deaths due to culling is estimated to be 0.97. For 2015–2020, the number of red deer reported to the game bag statistics decreased by approximately 2.9% per year, implying an annual growth rate of 0.971. We assume that the longevity of red deer in western Jutland is similar to a measure obtained for a population in eastern Jutland for the period between 2008 and 2012. Thus, the longevity of red deer is 3.52 years for females and 2.30 years for males, implying an average longevity of 2.91 years (see Sunde and Haugaard 2014). Now, we can use $N=CL\lambda /\eta$ to calculate a population of red deer in the western and central parts of Jutland on 14,144 individuals before the hunting season and 10,527 individuals after the hunting season. The browsing damage on agricultural crops mainly occurs during spring and summer, and therefore, it is reasonable to use the pre-hunting season population in the calculations of the marginal browsing damage costs (see Olaussen and Skonhoft, 2011). By using the total browsing damage cost and the population of red deer, we can calculate the marginal browsing damage cost.^4^ According to Gill et al. (2000) the marginal damage cost is linear in the size of red deer, so we can distribute the marginal browsing cost to our sex and age classes using the weight in Table 3.

### Constant Reflecting the Marginal Traffic Damage Costs

To identify the constant reflecting the marginal traffic damage costs for red deer in Denmark, we undertake an unadjusted benefit transfer. Specifically, we use a measure from COWI (2019), who reports the costs (defined as cost of material and salaries) of insurance claims for repairing motor vehicles involved in collisions with deer in Denmark in 2019. COWI (2019) reports an average cost per individual involved in an accident in Denmark but this cost covers collisions with all species of deer (roe, red, sika and fallow deer) and the distribution of various species is unknown. Thus, we have chosen to use the number reported by COWI (2019) as a measure for constant reflected the marginal traffic damage cost of red deer.^5^ According to Gren and Jagerbrand (2019) this damage cost is linear in the weight of red deer implying that we can distribute the constant reflecting the marginal damage cost to our sex and age classes using weight observations from Table 3.

### Probability of a Vehicle Collision

To estimate the probability of one deer being involved in a vehicle collision in Denmark, we assume that this parameter is constant and identical for all sex and age classes. In 2018, a total of 12,000 vehicle collisions with deer were registered in Denmark (see COWI, 2019). In the same year, red deer were involved in 295 out of 6862 vehicle collisions with all deer species in Denmark, where specially educated hunters were called out to track or euthanize injured animals (see Mayer et al., 2021). Thus, we obtain that 515 individuals of red deer were involved in a registered vehicle collision in Denmark in 2018. In 2018, a total of 9745 red deer were registered in the national game bag statistics, and as in subsection “Marginal browsing damage costs”, we can use $N=CL\lambda /\eta$ to obtain a spring population of red deer on 28,387 individuals. This leads to a probability of a vehicle collision with one red deer of 0.0182. As not all vehicle collisions with red deer are reported, it is reasonable to assume that the true probability is higher than 0.0182. We therefore assume that the probability of a vehicle collision is 0.02.^6^

## Results

The main purpose of this section is to report the results of the CBA of various nonoptimal management strategies (nonoptimal population and harvest) for red deer in Denmark. However, to understand the outcome of the CBA for understanding these results, it is useful to summarize the results from the biological model and this is done in Section “Biological model”. In subsection “Cost‒benefit analysis”, we will describe and explain the results of the CBA. To explain the results, it is useful to repeat three major assumptions behind the analysis in this paper. First, the population, harvest and net benefit under each management strategy and for each sex and age class are assumed to be constant and in a steady-state equilibrium. Second, the initial population under each management strategy is scaled to consist of 1000 individuals implying that we analyze the composition of the population and harvest on various sex and age classes. Finally, the meat and recreational values are related to the harvest while the browsing and traffic damage costs depend on the population.

### Biological Model

The results for the population and harvest of each sex and age class under each strategy are reported in Table 4.

**Table 4:** Results for the annual population and harvest for each sex and age class under each nonoptimal strategy, number of individuals

	Strategy				
	1	2	3	4.A	4.B
	Free harvest	Trophy hunting	Maximum harvest	Natural demographic population composition	Natural demographic population composition
**Sex and age-class**	**Population in the spring**				
Female calves	206	116	295	202	190
Female yearlings	134	75	83	101	95
Adult females	532	299	677	510	480
Male calves	206	116	295	202	190
Male yearlings	134	75	83	101	95
Young stags	172	217	56	171	185
Near-mature stags	25	204	53	69	95
Mature stags	4	130	50	47	50
**Sex and age-class**	**Harvest**				
Female calves	69	39	208	93	87
Female yearlings	25	14	7	16	15
Adult females	100	55	66	82	77
Male calves	69	39	208	93	87
Male yearlings	45	0	63	26	19
Young stags	70	1	0	44	36
Near-mature stags	12	1	0	18	19
Mature stags	2	63	16	12	20

As indicated in Table 4, the harvest of female and male calves is higher under strategy 3 (maximum harvest) than under the other strategies. The harvest of male yearlings, young stags and near-mature stags is close to zero under strategy 2 (trophy hunting), while the harvest of young stags and near-mature stags is approximately zero under strategy 3. Furthermore, under strategy 2, the harvest of female and male calves is lower than under the other strategies, while the harvest of mature stags is higher. From Table 4, we also see that the harvest of female yearlings and adult females is higher under strategy 1 (free hunting) than under the other strategies. When comparing strategies 4.A and 4.B (natural demographic population compositions), males are harvested at an older age in the latter case. The explanation for this result is that we assume differentiated hunting mortality rates for stags under strategy 4.B.

Table 4 also indicates that the population of female and male calves is higher under strategy 3 than under the other strategies. Furthermore, the population of adult females is higher under strategy 3 than under strategy 1, while the opposite holds for female yearlings, male yearlings and young stags. In addition, the population of near-mature stags and mature stags are lower under strategy 1 than under the other strategies, while the population of young stags, near-mature stags and mature stags are highest under strategy 2. When comparing strategies 4.A and 4.B, the population of females are higher in the former case, while the population of young stags, near-mature stags, and mature stags are higher in the latter case.

### Cost-benefit Analysis

In subsection “Benchmark case”, we will present the results of the CBA with benchmark marginal values and costs while the outcome of the sensitivity analyses are described in subsection “Sensitivity analyses”.

#### Benchmark Case

In Table 5 we report the results for the total values and costs with the benchmark parameter estimates (see Table 3).

**Table 5:** Results for the total annual values and costs for each sex and age class under each nonoptimal strategy, benchmark marginal values and costs, DKK

	Strategy				
	1	2	3	4.A	4.B
	Free harvest	Trophy hunting	Maximum harvest	Natural demographic population composition	Natural demographic population composition
**Total meat value**	539,930	313,982	554,301	496,565	472,355
Meat value, female calves	52,971	29,559	159,701	71,381	66,809
Meat value, female yearlings	29,847	16,572	8656	19,263	18,082
Meat value, adult females	147,114	81,685	97,180	121,883	114,412
Meat value, male calves	56,290	31,411	169,708	75,854	70,996
Meat value, male yearlings	57,985	O	80,628	33,356	23,973
Meat value, young stags	160,011	2272	0	99,451	82,417
Meat value, near-mature stags	31,636	2015	0	46,685	48,884
Meat value, mature stags	4076	149,852	38,427	28,692	46,782
**Total recreational value**	1,498,444	961,638	1,939,820	1,462,150	1,397,450
Recreational value, female calves	220,044	122,788	663,405	296,520	277,530
Recreational value, female yearlings	84,343	46,829	24,462	54,433	51,096
Recreational value, adult females	358,329	198,963	236,705	296,872	278,677
Recreational value, male calves	220,044	122,788	663,405	296,520	277,530
Recreational value, male yearlings	168,380	0	234,134	96,863	69,614
Recreational value, young stags	361,292	5130	0	224,553	186,092
Recreational value, near-mature stags	73,524	6117	0	108,501	113,610
Recreational value, mature stags	12,487	452,023	117,710	87,889	143, 302
**Total browsing damage cost**	1,413,255	1,596,790	1,524,006	1,488,731	1,508,643
Browsing damage cost, female calves	116,135	65,209	166,220	114,025	107,175
Browsing damage cost, female yearlings	120,192	67,487	74,103	90,775	85,322
Browsing damage cost, adult females	582,244	326,924	740,484	558,383	524,839
Browsing damage cost, male calves	124,990	70,180	178,892	122,718	115,346
Browsing damage cost, male yearlings	127,152	71,394	78,394	96,032	90,263
Browsing damage cost, young stags	288,146	363,167	93,590	286,969	311,026
Browsing damage cost, near-mature stags	47,971	395,212	101,848	134,455	184,125
Browsing damage cost, mature stags	6425	237,216	90.475	85,374	90,547
**Total traffic damage cost**	622,642	703,567	671,431	655,911	664,690
Traffic damage cost, female calves	51,161	28,727	73,225	50,232	47,214
Traffic damage cost, female yearlings	52,967	29,741	32,657	40,004	37,601
Traffic damage cost, adult females	256,539	144,044	326,260	246,025	231,246
Traffic damage cost, male calves	55,037	30,903	78,772	54,036	50,790
Traffic damage cost, male yearlings	56,006	31,447	34,530	42,298	39,757
Traffic damage cost, young stags	126,953	160,019	41,238	126,444	137,045
Traffic damage cost near-mature stags	21,138	174,146	44,878	59,246	81,133
Traffic damage cost, mature stags	2832	104,542	39,873	37,625	39,904
**Total net benefit**	2477	−1,024,737	296,684	−185,927	−303,527

From Table 5, we see that the total net benefit is positive and highest under strategy 3 (maximum harvest), implying that this management strategy should be implemented from an economic point of view. This result can be explained by the fact that the total meat and recreational value is higher under this strategy than under the others. Specifically, the meat and recreational values of female and male calves are highest under strategy 3 because the harvest of female and male calves are higher than under the other strategies (see Table 4).

For strategy 1 (free harvest) the results indicate that the total net benefit is small but positive because the total browsing and traffic damage costs are lower than under the other strategies. This result occur because the browsing and traffic damage costs of near-mature stags and mature stags are lowest under strategy 1 and based on Table 4 this can be explained by the fact that the populations of near-mature stags and mature stags are lowest.

Table 5 also indicate that the total net benefit under strategy 2 (trophy hunting) is relatively large and negative. Thus, if strategy 2 is the only management strategy, we shall extinct the population of red deer. Main drivers behind this result is that: (a) the total meat and recreational value is lowest under strategy 2: and (b) the total browsing and traffic damage costs are highest under strategy 2. (a) arise because the total meat value, recreational value and harvest for female and male calves are lowest while (b) occur because the total browsing damage cost, traffic damage cost and populations of young stags, near-mature and mature stags is highest.

From Table 5, we also observe that both strategies 4.A and 4.B (natural demographic population compositions) generate a reasonable small negative net benefit. Thus, if strategies 4.A and 4.B are the only management options, the red deer population shall be driven to extinction. However, the net benefit is slightly higher under strategy 4.A than under strategy 4.B which can be explained by the facts that: (a) the total meat and recreational value is slightly higher under strategy 4.A because males are harvested at an older age under strategy 4.B; and (b) the total browsing and traffic damage costs are slightly lower under strategy 4.A because the population of young stags, near-mature stags and mature stags is lower.

#### Sensitivity Analyses

When reporting the results of the sensitivity analyses, we focus on the total annual net benefit under each nonoptimal strategy. Furthermore, we will use the sensitivity analysis to discuss the robustness of our results with benchmark marginal values and costs.^7^ As mentioned in Section “Parameterization for red deer in Denmark”, we have chosen to vary the parameter estimates for the same marginal value or cost simultaneously by ± 50% in the sensitivity analyses. However, as indicated in Eq. (12), the marginal meat value (${mw_{i}}$) is defined as the meat price (${m}$) times the weight of red deer (${w_i}$). Thus, varying the price or weight of red deer by ± 50% gives an identical net benefit. Furthermore, instead of creating an upper and lower bound for the price or weight, we will operate with such bounds for the marginal meat values (${mw_{i}}$). In a similar way, we will operate with an upper and lower bound for the marginal traffic damage costs (${t_{i}\alpha}$) instead of such bounds for the constant reflecting the marginal traffic damage costs (${t_{i}}$) or the probability of one red deer being involved in a traffic incident (${\alpha}$).

The results of the sensitivity analyses are reported in Table 6.

**Table 6:** Results for the total annual net benefits under each nonoptimal strategy, upper and lower bound for marginal values and costs, DKK

Strategy		Lower bound				Benchmark value	Upper bound			
		Marginal meat values	Marginal recreational values	Marginal browsing damage costs	Marginal traffic damage costs		Marginal meat values	Marginal recreational values	Marginal browsing damage costs	Marginal traffic damage costs
**1**	**Free harvest**	−267,488	−746,745	709,104	313,798	2477	272,442	751,699	−704,150	−308,844
**2**	**Trophy hunting**	−1,183,000	−1,506,899	−228,352	−674,963	−1,024,737	−870,094	−546,595	−1,825,142	−1,378,531
**3**	**Maximum harvest**	27,023	−667,007	1,067,159	640,872	296,684	583,290	1,277,320	−456,846	−30,559
**4.A**	**Natural demographic population composition**	−434,209	−917,002	558,439	142,029	−185,927	62,356	545,148	−930,292	−513,882
**4.B**	**Natural demographic population composition**	−539,705	−1,002,252	450,794	28,818	−303,527	−67,349	395,198	−1,057,848	−635,872

Table 6 indicate that the ranking of the strategies from the benchmark case holds for all parameter variations. The explanation for this result is that parameter variations affect the net benefit under all strategies in a similar way. Furthermore, for the lower bound of the marginal recreational values, the upper bound of the marginal browsing damage costs and the upper bound of the marginal traffic damage costs, the net benefit under all strategies becomes negative. In this case the population of red deer shall be extinct under all strategies.

Concerning each individual strategy the total net benefit is positive under strategy 3 in the benchmark case but this net benefit becomes negative for the lower bound of the marginal recreational values, the upper bound of the marginal browsing damage costs and the upper bound of the marginal traffic damage costs. Under strategy 1, the total net benefit is also positive in the benchmark case, but it becomes negative for the lower bound of all marginal values and the upper bound of all marginal damage costs. In the benchmark case, the total net benefit is negative under strategy 2, and this result is highly robust in the sense that it holds for all parameter variations. Under strategy 4.A, the total net benefit is negative in the benchmark case but it becomes positive for the upper bound of all marginal values and the lower bound of all marginal damage costs. The total net benefit is also negative under strategy 4.B, but for the lower bound of the marginal browsing damage costs, the upper bound of the meat values and the upper bound of the marginal recreational value it become positive.

## Brief Summary and Discussion

The purpose of this paper is to undertake a CBA of alternative nonoptimal management strategies for red deer in Denmark. To capture the effect of various harvest strategies on the population of red deer, we have used a sex- and age-structured biological model. We operate with seven sex and age classes represented by female calves, male calves, female yearlings, male yearlings, adult females, young stags, near-mature stags and mature stags. In the CBA, we include meat values, recreational values, browsing damage costs and traffic damage costs, and these values and costs are assumed to differ for the various sex and age classes of red deer. The nonoptimal strategies consist of free harvest, trophy hunting, maximum harvest and natural demographic population compositions. We show that the maximum harvest strategy leads to a reasonably high positive total net benefit, while the strategy with free harvest yields a small positive net benefit. Furthermore, the trophy hunting strategy generates a large negative net benefit, while a small negative net benefit is obtained under the two strategies for natural demographic population compositions.

In relation to the results of the CBA, at least six issues are useful to discuss. First, in the biological population model, we have assumed a steady-state equilibrium implying that the population, harvest and net benefit is constant over time. However, it can be argued that we should have used a dynamic model by taking adjustment paths toward a steady-state equilibrium into account (see, e.g., Clark, 1990). Within a dynamic model, the population and harvest of red deer may change over time, implying that the total net benefit becomes nonconstant. Thus, we must calculate the present value of the current and future net benefit, and therefore, discounting shall be taken into account (see, e.g., Mishan and Quah, 2021). Furthermore, we must compare the management strategies by considering the present value of the current and future net benefits. However, a dynamic sex- and age-structured model will often generate strange and/or unrealistic adjustment paths even under very strict assumptions (see, e.g., Quass and Tahvonen, 2019). Thus, as a simplification it seems reasonable to assume a steady-state equilibrium.

Second, it can be argued that our policy conclusions would change if the marginal values and costs differ significantly from the benchmark parameter estimates. Specifically, when estimating the marginal values and costs for red deer in Denmark we have imposed a number of strict assumptions. This may imply that our measures differ from the true marginal values and costs and this may affect our results. However, we have conducted sensitivity analyses and here we have chosen a very large parameter variation on ± 50%. Thus, it seems reasonable to argue that the true marginal values and costs lies within the span generated by this parameter variation. Furthermore, the ranking of the strategies is unaffected by parameter variations because a change in a marginal value or cost will affect the net benefit under all strategies in a similar way. Thus, it seems reasonable to argue that our results hold for alternative parameter estimates for the marginal values and costs.

Third, a related issue is that we have used simple methods such as benefit transfer to estimate the marginal values and costs. With benefit transfer, we use a value or cost from another study on our case represented by red deer in Denmark and for obvious reasons, this approach is subject to a high degree of uncertainty. To minimize this uncertainty, we have used other studies for red deer in Denmark to undertake benefit transfers. An alternative is to undertake primary valuation studies using either stated or revealed preference valuation methods which would generate more precise parameter estimates (see Freeman et al., 2014). However, undertaking a primary valuation study is outside the scope of this paper, and to address the issue with uncertainty under benefit transfer, we have conducted sensitivity analyses by varying the marginal values and costs with ± 50%.

Fourth, we have obtained that trophy hunting yields a large and negative total net benefit while Naevdal et al. (2012) have shown that trophy hunting may be an optimal strategy for moose in Scandinavia. In Naevdal et al. (2012) the population of moose is structured as calves, yearlings, adult females and adult males. For calves, yearlings and adult females the net benefits are defined using a constant marginal meat value while a trophy value arise for adult males. The trophy value is captured by a demand function that depends on the harvest and population of adult males. This implies that the trophy value becomes higher than the meat value which explains why trophy hunting can be an optimal strategy. In our paper, the trophy value is captured by the marginal recreational value for near-mature stags and mature stags and we obtain a large negative net benefit under trophy hunting because: (a) the total meat value and recreational value is lowest under trophy hunting because the total meat value and recreational value for female and male calves is lowest; and (b) the total browsing and traffic damage costs is highest under trophy hunting because the total browsing damage cost and traffic damage cost for young stags, near-mature and mature stags is lowest. (a) and (b) can explain the differences in the results for trophy hunting compared to Naevdal et al. (2012).

Fifth, we have assumed constant marginal values and costs. This implies that strategies with extreme values for the population and harvest become relatively more desirable (see Freeman et al., 2014). Thus, constant marginal values and costs may explain why the with maximum harvest and free harvest strategies yield a positive net benefit, while we obtain a large negative net benefit under trophy hunting strategy. An alternative is to assume positive and increasing marginal costs in the population and positive but decreasing marginal values in the harvest. These assumptions imply that strategies with nonextreme values for the population and harvest become relatively more beneficial (see Freeman et al., 2014). Thus, the strategies for natural demographic population compositions tend to become more desirable with nonconstant marginal values and costs.

Finally, we have conducted a CBA of several nonoptimal management strategies. These strategies imply that we accept an efficiency loss in the sense that a nonoptimal population and harvest of red deer are reached (see, e.g., Hanley et al., 1997). If we want to avoid this efficiency loss, we should target an economically optimal population and harvest, and this approach has been used in several papers on the management of red deer (see Skonhoft et al., 2013, Smart et al., 2008 and Rondeau and Conrad, 2003). However, it is well known, that targeting an optimal population and harvest of an animal species is not always politically feasible (see, e.g., Svensson et al., 2015). Therefore, it seems well-justified to conduct a CBA of nonoptimal management strategies.

## Supplementary information

Demographic model for red deer - description

Red deer demographic models 2024-07-01

## Footnotes

1 Scottish Government (2020) have reported prices on red deer meat in Scotland while Gaviglia et al. (2018) estimate prices in Italy. By making these prices comparable with our prices the price on red deer meat in Scotland and Italy is between 18 DKK/kg and 22 DKK/kg. Thus, our price on red deer meat in Denmark is in line with the prices in comparable countries.

2 The weight differ for various red deer populations in Denmark, but the relative difference in weight between sex and age classes is approximately identical (see Sunde and Haugaard, 2014). Thus, the weight of red deer reported in Table 3 generalize to for other regions in Denmark.

3 The recreational value in Jensen et al. (2022) covers two hunting days and the possibility of bagging one individual. However, many hunters probably use more than two days to bag one individual implying that our measure tends to be a lower bound for the true marginal recreational value. Furthermore, recreational and existence values of the general public are not taken into account which also implies that our measure tends to be a lower bound for the true value.

4 Due to lack of data our marginal damage cost measure may represent a lower bound for the true marginal damage cost. Furthermore, we have assumed that farmers, who did not respond to the survey in Løbner (2021), have a browsing damage cost on zero. In reality, many of these farmers have a positive browsing damage costs which also imply that our measure tends to be a lower bound for the true measure. On the other hand, farmers may have an incentive to exaggerate the browsing cost if they believe that this can affect future compensations implying that our measure tends to become an upper bound for the true measure.

5 This damage cost does not include physical or psychological injuries caused by collisions with deer, implying that it tends to be a lower bound for the true measure. However, the cost measure covers all deer species, which tends to imply that it is an upper bound for the true costs.

6 In comparison, the probability was estimated to be between 0.021 and 0.065 for four different deer species in Finland (see Niemi et al., 2015). Furthermore, the probability of a vehicle collision is estimated to be between 0.01 and 0.02 for moose in Sweden (see Seiler, 2005). Thus, our measure for the probability is in line with comparable estimates form similar countries.

7 An alternative is to focus on numerical comparative static results and thereby investigate whether the results of the sensitivity analyses can be explained in a straightforward way. As indicated in Table 6 the numerical comparative static results are identical under all strategies and we have that the total net benefit increases (decreases) with an increase (decrease) in the marginal values and a decrease (increase) in the marginal damage costs. Based on the definition of the net benefit in Eq. (12), these results seems reasonable.
