# Supplementary material for: A Cost-Benefit Analysis of Alternative Management Strategies for Red Deer in Denmark
Source: Environ Manage. 2024 Aug 7;74(4):648–63. doi: 10.1007/s00267-024-02023-y (PMC11393284; doi:10.1007/s00267-024-02023-y)
Supplement: Supplementary file 1 — Demographic model for red deer - description [file 267_2024_2023_MOESM1_ESM.pdf]

Reference and contact person

Suggested reference: Sunde, P. (2024). Sex- and age structured population model developed for analysis of red deer harvest strategies. Published as electronic supplementary material to Jensen, F., Lundhede, T. & Sunde, P. (2024): A Cost–Benefit Analysis of Alternative Management Strategies for Red Deer in Denmark. Environmental Management

Correspondence: Peter Sunde, Aarhus University, Department of Ecoscience, C.F. Møllers Allé 8, 8000 Aarhus C, Denmark. E-mail: psu@ecos.au.dk

Description and principles of the model

The demographically structured population model is structured like a sex-specific lifetable, where the demographic composition of the population and number of individuals dying is the emergent pattern of a) age-specific fecundity of females, b) sex composition of recruits entering the population (default value = 0.5, representing an even sex ratio), and c) age-specific mortality of males and females (Caughley, 1977. Analysis of Vertebrate Populations. Blackburn Press, New Jersey.).

The current model is constructed to model harvest scenarios of red deer in a managed Danish landscape (five different models are presented on each their sheet) but can in the principle be applied to other organisms with different sex and age specific mortality and fecundity with a maximum age of 24 years/time steps.

The number of recruits (calves) born into the population is defined by the number of adult females, multiplied with their age specific fecundity. In the five scenarios presented, we used Danish population data on the proportion of lactating hinds at the start of the hunting season, which is 1st September in Denmark, which is 0.57 for females at two years of age (third year of life) and 0.82 for females above two years of age (fourth year of life and older) (Sunde and Haugaard 2014: http://dce2.au.dk/pub/SR106.pdf). The calf-sex ratio before the beginning of the hunting season was assumed to be even (half of all recruits are females, the other half males).

Input parameters that specify the model is entered in cells with yellow background (all other cells are locked for editing). Output parameters are returned in cells with grey background. Please be aware that not all parameter values or combinations of parameter are useful. For example, annual mortality rates must be within the range between 0 (no mortality) to 1 (all in age class die). The same is the case for the proportion of recruits that are females. To ensure that the population is at a static, steady-state equilibrium (the number of deaths equaling the number of births, and a constant sex and age distribution between years), be careful to only use combinations of age specific female fecundity and mortality rates that resulted in deaths and births being perfectly balanced (l = 1.00). Therefore, make sure that the annual population

growth (multiplication) value (calculated over 100 years) in cell B6 is as close to 1 as possible (a deviation of plus/minus 0.01 is acceptable)

Specific entities of the model and how to use it

The model is designed to model a harvested population with a limited non-harvesting mortality (traffic deaths, diseases, predators etc.). To separate between harvest and non-harvest mortality, it is therefore possible to specify a non-harvest mortality so that non-harvest deaths are subtracted from the total mortality (remaining deaths are then attributed to harvest). As non-harvest mortality is a component of the total mortality, make sure that the non-harvest mortality rate does not exceed the total sex- and age specific mortality.

It is possible to differentiate non-harvest mortality rate between sex and age classes by using the relative selection (or hazard) matrix (cells K9-L33). Based on the relative magnitude of the numbers in the matrix, non-harvest mortality will be distributed selectively amongst the different sex and age classes. With the default values of 1 (or any other positive number that is the same for all sex and age classes) all sex and age classes are suffering the same annual mortality rate as entered in cell B4 for the spring population. If (say) female yearlings (cell K10) are known to suffer twice as high non-harvest mortality than all other sex and groups, you just enter a value that is twice as high for this group than for all other groups and the deaths will be allocated between groups in relation to this differentiated mortality. You can enter different selection values for all 50 sex and age classes.

Input parameters (cells): description

Female proportion of calves (B2): The proportion of calves recruited to the population that are females (default = 0.5 [even sex ratio])

Population size (spring) (B3): Number of individuals in the spring population (before the calves of the year are born).

Mean annual mortality rate: other reasons than harvest (relative to spring population size) (B4): The proportion of the spring population dying every year of reasons than hunting (e.g. traffic, disease, accidents, predation etc.). The total number of deaths per year in the population as result of this 'background' mortality rate will appear in cell B14.

Selection values for other death causes than harvest (K9-K33 [females, age 0-24]), L9-L33 [males, age 0-24])**:** If the mean mortality rate due to other reasons than hunting differs between sex and age, the relative hazard rate can be entered here. Default is 1 for all sex and age groups which means that all

have the same risk of dying of other reasons than hunting. If a sex/age group is provided with a value of 2 relative to 1 for all other classes, this group will suffer twice as high mortality rate due to non-hunting reasons than all other groups.

Annual mortality rates (E9-E33 [Females, age 0-24]); G9-G33[males, age 0-24]): Total annual mortality rate for each sex- and age class (number between 0 [no mortality] and 1 [all in age class die]). Must not be lower than the age/sex specific mortality rate due to other reasons than hunting

Overall output parameters (cell reference): description

Mean annual mortality rate: other reasons than harvest (relative to autumn population size) (B5): The proportion of the autumn population (spring population +recruits of the year) dying every year of reasons than hunting (e.g. traffic, disease, accidents, predation etc.). The total mortality rate must be at least as large as the non-harvest mortality rate (indicated by an "OK" in the cell to the right). Otherwise, the message "Error: non-hunting mortality rate exceeds total mortality rate!" is returned.

Lampda (annual growth [multiplication] rate) (B6): Mean annual multiplication rate calculated over 100 years. To maintain a stable population, the value should be as close to 1 as possible.

Population size in spring (B7): Total number of individuals in the spring population (same as the input parameter entered in B3).

Number of males in spring (B8)**:** Total number of male individuals in the spring population.

Number of females in spring (B9): Total number of female individuals in the spring population.

Proportion of males in spring population (B10): The proportion of the spring population comprised by males.

Calves born per year (B11): Number of calves born/recruited per year.

Annual no of deer dying every year (B12): Total annual number of deer dying every year (harvested + other deaths cause).

Annual no of deer dying by other reasons than harvest (B13): The total number of deer in the population dying annually by other reasons than harvest. Calculated as spring population size (B3) multiplied by mean annual mortality rate due to other reasons than harvest (B4).

Number of individuals harvested (B14): Number of individuals harvested (Total number of deaths [B12] - Number of individuals dying of other reasons than harvest [B13]).

population size on 1. sept (B15): Spring population size [B7] + number of calves recruited into population during summer [B11]).

proportion of stags 8+ years in spring population (B16): Number of males at 8+ year of age / Total number of males in spring population

hinds 1+ year: stags 8+ year ratio in September (B17): Number of (fertile) females per 8+ year old stag during at 1 September (start of rutting season)

hind:stag ratio (1+ year), September (B18): Number of (fertile) females per 1+ year old stag during at 1 September (start of rutting season)

hind:stag ratio (1+ year), spring (B19): Number of 1+ old hinds per 1+ year old stag in spring population

Number of stags harvested at 5+years of age (B20): Number of 5+ year old stag harvested per year

Number of stags harvested at 8+year of age (B21)**:** Number of 8+ year old stag harvested per year

Sex and age specific output parameters (cell reference): description

Females, survival to age x, lx (N9-N33): The proportion of FEMALES surviving to enter age class 0-24, respectively.

Females, rel. age composition (within sex), 1. Sep. (O9-O33): proportion of individuals in the FEMALE segment of population belonging to age class x (0-24) on 1 Sept. (sums up to 100%).

Females, rel. age composition (within sex), spring (P10-P33): proportion of individuals in the FEMALE segment of population belonging to age class x (1-24) in spring (sums up to 100%)

Males, survival to age x, lx (R9-R33): The proportion of MALES surviving to enter age class 0-24.

Males, rel. age composition (within sex), 1. Sep. (S9-S33): proportion of individuals in the MALE segment of population belonging to age class x (0-24) on 1 Sept. (sums up to 100%).

Males, rel. age composition (within sex), spring (T10-T33): proportion of individuals in the MALE segment of population belonging to age class x (1-24) in spring (sums up to 100%).

Population composition (1 Sept), Proportional, F and M (V9-W33): Proportion of population at 1 Sep (start of hunting season) comprised by different sex (F: females, M: males) and age groups (0-24 years) (sums up to 100%).

Population composition (1 Sept), Number of individuals, F and M (X9-Y33): Number of individuals in population at 1 Sep (start of hunting season) divided on sex (F: females, M: males) and age groups (0-24 years) (sums up to total population size).

Total number of deaths per year (Z9-AA33): Total number of deaths per year divided on sex (F: females, M: males) and age groups (0-24 years) (sums up to total number of deaths per year).

Number of calves produced per age class (AC11-AC33): The product of the number of females per age class (2-24) and their age-specific fecundity.

Non-harvest mortality, Sex and age composition (AE9-AF33): Proportion of non-harvest mortality cases, divided on sex and age groups (sums up to 100%).

Non-harvest mortality, Number per year (AG9-AH33): Number of non-harvest mortality cases per sex and age groups per year (sums up to the total number of non-harvest death in the population per year).

Non-harvest mortality, Proportion of all deaths (AL9-AM33): the proportion of all deaths within a given sex and age class that is attributable to other reasons than harvest.

Standing crop, Population size (1 Sept) F and M (AP9-AQ33): Number of individuals of each sex and age group (0-24) in population by 1 September (sums up to total population size by 1 September).

Harvested, Number harvested, F and M (AS9-AT33): Number of individuals from each sex and age group harvested per years (sums up to total harvest size).

Non-harvest mortality, Cause-specific mortality rate per year (AU9-AV33): the number of individuals within each sex and age class dying of other reasons than harvest during a year.
